# Myelin-Specific microRNA-23a/b Cluster Deletion Inhibits Myelination in the Central Nervous System during Postnatal Growth and Aging

**DOI:** 10.3390/genes15040402

**Published:** 2024-03-25

**Authors:** Shigeki Ishibashi, Naosuke Kamei, Yuji Tsuchikawa, Toshio Nakamae, Takayuki Akimoto, Shigeru Miyaki, Nobuo Adachi

**Affiliations:** 1 Department of Orthopaedic Surgery, Graduate School of Biomedical and Health Sciences, Hiroshima University, Hiroshima 734-8551, Japan; rugbyshige@gmail.com (S.I.); toshinakamae623813@yahoo.co.jp (T.N.); miyaki@hiroshima-u.ac.jp (S.M.); nadachi@hiroshima-u.ac.jp (N.A.); 2 Orthopedics and Micro-Surgical Spine Center, Hiroshima City North Medical Center Asa Citizens Hospital, Hiroshima 731-0293, Japan; tsuchiriver@gmail.com; 3 Faculty of Sport Sciences, Waseda University, Tokorozawa 359-1192, Japan; axi@waseda.jp; 4 Medical Center for Translational and Clinical Research, Hiroshima University Hospital, Hiroshima 734-8551, Japan

**Keywords:** microRNA, cluster, myelin, oligodendrocyte, brain, spinal cord, aging

## Abstract

Microribonucleic acids (miRNAs) comprising miR-23a/b clusters, specifically miR-23a and miR-27a, are recognized for their divergent roles in myelination within the central nervous system. However, cluster-specific miRNA functions remain controversial as miRNAs within the same cluster have been suggested to function complementarily. This study aims to clarify the role of miR-23a/b clusters in myelination using mice with a miR-23a/b cluster deletion (KO mice), specifically in myelin expressing proteolipid protein (PLP). Inducible conditional KO mice were generated by crossing miR-23a/b cluster^flox/flox^ mice with *PlpCre-ERT2* mice; the offspring were injected with tamoxifen at 10 days or 10 weeks of age to induce a myelin-specific miR-23a/b cluster deletion. Evaluation was performed at 10 weeks or 12 months of age and compared with control mice that were not treated with tamoxifen. KO mice exhibit impaired motor function and hypoplastic myelin sheaths in the brain and spinal cord at 10 weeks and 12 months of age. Simultaneously, significant decreases in myelin basic protein (MBP) and PLP expression occur in KO mice. The percentages of oligodendrocyte precursors and mature oligodendrocytes are consistent between the KO and control mice. However, the proportion of oligodendrocytes expressing MBP is significantly lower in KO mice. Moreover, changes in protein expression occur in KO mice, with increased leucine zipper-like transcriptional regulator 1 expression, decreased R-RAS expression, and decreased phosphorylation of extracellular signal-regulated kinases. These findings highlight the significant influence of miR-23a/b clusters on myelination during postnatal growth and aging.

## 1. Introduction

Myelination in the central nervous system (CNS) is crucial for the efficient transfer of information between neurons and for providing metabolic support to axons [1,2]. Oligodendrocytes are responsible for producing myelin, a lipid-rich sheath essential for rapid signal transduction along axons [3,4]. The significance of myelin is further underscored by the functional impairments observed in demyelinating diseases, such as multiple sclerosis [5]. Myelination is a complex, multistep process encompassing the proliferation of oligodendrocyte progenitor cells (OPCs), their differentiation, and the subsequent formation of myelin sheaths around axons [6,7]. Recent advancements have increasingly highlighted the pivotal role of microribonucleic acids (miRNAs) in the regulation of myelination [8,9,10,11,12,13,14].

MicroRNAs (miRNAs) are short (~22 nucleotides) noncoding RNAs recognized as key regulators of gene expression [15]. Many miRNAs maintain cellular activity by regulating protein translation from genes through tissue-specific expression. The deletion of Dicer1, an enzyme essential for miRNA maturation, significantly inhibits oligodendrocyte differentiation and myelination, underscoring the critical role of miRNAs in CNS myelination [16]. In addition, clustered miRNAs regulate molecular pathways in a complementary manner, suggesting that the deletion of one miRNA in a cluster may be compensated by the action of other miRNAs in the same cluster [17]. This compensatory mechanism emphasizes the need to investigate miRNAs on a cluster-specific basis to fully comprehend their biological functions.

Although miR-23a and miR-27a are part of the miR-23-27-24 cluster, they reportedly exert opposing effects on myelination regulation in the CNS [12,13]. In our previous study, mice with a global miR-23a/b cluster knockout (KO) from embryonic life, comprising miR-23a-27a-24-2 (miR-23a cluster) and miR-23b-27b-24-1 (miR-23b cluster), showed hypoplastic myelin sheath formation in the brain and spinal cord [14]. However, due to the widespread expression of miR-23a/b clusters in various tissues, including the lungs, skeletal muscle, liver, and CNS, studies using global miR-23a/b cluster KO mice have been unable to delineate tissue-specific mechanisms [18,19]. Moreover, the role of miR-23a/b clusters in postnatal growth and aging could not be evaluated, given the deficiency of miR-23a/b clusters from the developmental stage.

In the present study, we focused on the myelin proteolipid protein (PLP), which is specifically expressed in myelin, and generated inducible KO mice by deleting miR-23a/b clusters in cells expressing the *Plp* gene, facilitated by tamoxifen administration. This study aims to elucidate the role of miR-23a/b clusters in CNS myelination by examining the effects of myelin-specific miR-23a/b cluster deletion during postnatal growth and subsequent aging in miR-23a/b cluster KO mice.

## 2. Materials and Methods

### 2.1. Ethics Statement

This study was conducted with strict adherence to the Guidelines for Proper Conduct of Animal Experiments set by the Science Council of Japan. All animal care and handling procedures were approved by the Committee of Research Facilities for Laboratory Animal Sciences of the Graduate School of Biomedical Sciences, Hiroshima University (Approval No. A18-134). This ensured the ethical and humane treatment of all animals used in this study.

### 2.2. Animals and Genotyping

Myelin-specific miR-23a/b cluster KO mice were generated by crossbreeding *Plp cre*-driver mice (Cyagen, Santa Clara, CA, USA) [20] with miR-23a/b clusterfloxed mice on a C57BL6/J background [18,21]. In this study, genetically modified *Plp Cre^ERT2^*;miR-23a/b cluster mice—a model designed for tamoxifen-induced gene KO—were used, allowing for controlled manipulation of gene expression during specific developmental stages [22,23]. Mice were individually housed under controlled conditions, including a 12 h light/dark cycle, with room temperature maintained at 23 ± 1 °C and humidity levels between 40 and 60%. These conditions were strictly monitored to ensure a stable environment.

Tamoxifen (S1238; SelleckChem, Houston, TX, USA), dissolved in corn oil, was administered intraperitoneally to induce gene KO. Starting from seven days of age, mice received 66 µg/g body weight tamoxifen for seven consecutive days. At 10 weeks of age, the dosage was increased to 100 µg/g body weight and administered for eight consecutive days. Control mice were administered an equivalent volume of corn oil without tamoxifen, following the same dosing schedule as that for KO mice. Mice treated with tamoxifen or corn oil at 10 days of age were sampled at 10 weeks of age, and those treated at 10 weeks of age were sampled at 12 months of age for evaluation.

Genotyping was performed using genomic deoxyribonucleic acid extracted from the tail tip, as previously described [18,19]. The KO mice were specifically identified using PCR with the primers 5′-GCA TTA CCG GTC GAT GCA ACG AGT GTA GAG-3′ and 5′-GAG TGA ACG AAC CTG GTC GAA ATC AGT GCG-3′.

### 2.3. Real-Time PCR

The expression of miRNAs in miR-23a/b clusters (miR-23a, miR-23b, miR-27a, miR-27b, and miR-24) in 10-week-old and 12-month-old control and KO mice was evaluated using real-time polymerase chain reaction (PCR). The mice were anesthetized with isoflurane and transcardially perfused with 20 mL of sterile RNase-free phosphate-buffered saline (PBS). The spinal cord white matter and brain corpus callosum were collected from six control and six KO mice at 10 weeks and 12 months of age. Spinal cord gray matter, brain gray matter, liver, lungs, and muscles were collected from four mice per group. Total RNA was extracted from these tissues using ISOGEN (Nippon Gene, Tokyo, Japan), according to the manufacturer’s instructions. To synthesize first-strand complementary deoxyribonucleic acid, total RNA was reverse-transcribed using the TaqMan MicroRNA Reverse Transcription Kit (Thermo Fisher Scientific, Waltham, MA, USA). The expression of miR-23a (000399), miR-23b (000400), miR-27a (000408), miR-27b (000409), and miR-24 (000402) was analyzed using TaqMan MicroRNA Assays (Thermo Fisher Scientific). Real-time PCR was performed in 96-well plates using the Applied Biosystems StepOne Real-time PCR System (Thermo Fisher Scientific). The expression of each miRNA was normalized to that of the U6 small nuclear RNA (001973).

### 2.4. Behavioral Assessments

Behavioral assessments were performed on 10-week-old and 12-month-old control and KO mice. Motor function tests were performed using methods described in previous reports, including the hanging wire and balance beam tests [14,24,25]. The hanging wire test assessed overall subacute muscle function and coordination. In this test, a 2 mm thick metal wire was fixed to two 35 cm high vertical stands. Mice were hung by their forelimbs in the center of the wires, and the time taken for them to fall was measured (up to 3 min). The balance beam test assessed motor coordination and equilibrium. A beam that was 6 mm wide, 80 cm long, and 25 cm high was used (10 cm front and back margins in addition to the 80 cm length. The time required for each mouse to cross the beam was recorded. A brightly illuminated starting platform and nonilluminated escape box were placed at each end of the beam. For each test, measurements were performed three times per mouse, and the average value was used for evaluation. All experiments were conducted in the dark. The experimenters were blinded to the genotype of each mouse during behavioral testing and subsequent analysis.

### 2.5. Electron Microscopy

Electron microscopy was used to evaluate myelination in the spinal cords of 10-week-old and 12-month-old control and KO mice. Mice were anesthetized with isoflurane, perfused briefly with PBS, and then with 2% glutaraldehyde in 0.1 M cacodylate (pH 7.2). The spinal cord at the level of the tenth thoracic vertebra was removed and fixed overnight at 4 °C in a fresh fixative solution. The tissues were washed in PBS, fixed for 2 h in 1.5% OsO4 in PBS, dehydrated in a graded ethanol solution, infiltrated with propylene oxide, and embedded in Epon. Semi-thin sections were stained with toluidine blue, and 70–80 nm thick sections were stained with 2% uranyl acetate and lead citrate. The ventral–lateral portion of the white matter in the spinal cord was used as the target region. The G-ratio of the axons in the target region was determined as the ratio of the diameter of the axon to the diameter of the myelin sheath associated with the axon [1]. Approximately 120–180 axons per mouse were analyzed in eight mice per group. Digitized and calibrated images were analyzed using ImageJ software (Version 1.54, National Institutes of Health, Bethesda, MD, USA).

### 2.6. Luxol Fast Blue Staining

The white matter of the brain and spinal cords of 10-week-old and 12-month-old control and KO mice were evaluated using Luxol Fast Blue staining. Mice were perfused transcardially with 20 mL of PBS, followed by 40 mL of a newly prepared 4% paraformaldehyde (PFA) solution. Whole brains and spinal cords at the level of the eighth thoracic vertebra were harvested, fixed in PFA for 8 h, and incubated at 4 °C in concentrated sucrose solutions (15% and 30%). The tissues were then immersed in an optimal cutting temperature compound (Sakura Finetek Japan, Tokyo, Japan), frozen, and stored at −80 °C. Fixed tissues were sectioned into 14 μm thick sections using a cryostat. Frozen sections were collected on slides, thawed, rehydrated in PBS, incubated with Luxol Fast Blue Solution (LBC-2-IFU; ScyTek Laboratories, Logan, UT, USA) in a sealed container for 10 h at 60 °C, washed with distilled water, and placed in 95% alcohol. The sections were then incubated for 10 min in 95% alcohol at 60 °C, followed by further incubation in a 0.05% lithium carbonate solution for 10 s and 70% alcohol solution for 20 s. These latter two steps were repeated until the gray and white matter could be clearly observed under the microscope. Twenty consecutive coronal sections of the splenium of the corpus callosum were obtained to measure its thickness. The thickness of the cerebral corpuscles was also measured in these 20 sections, and the maximum value was used for analysis. In the transverse sections of the spinal cord, the areas of the entire spinal cord and white matter were measured. The white matter ratio was calculated as the ratio of the white matter area to the entire spinal cord area. Five consecutive sections were used to calculate the white matter ratio, and the average value was used for analysis. Eight mice of each genotype were examined. ImageJ software (Version 1.54) was used to measure the lengths and areas.

### 2.7. Immunohistochemistry

The coronal sections of the corpus callosum and transverse sections of the spinal cord were immunostained for oligodendrocytes [26]. Transverse cryostat sections (16 μm) of the brain and spinal cord were fixed overnight in 4% PFA/PBS and cryoprotected overnight in 20% sucrose/PBS before sectioning, as previously reported. These sections were permeabilized with 0.3% Triton X-100, and nonspecific binding sites blocked with serum matching the species of the secondary antibody. That is, when anti-mouse IgG monoclonal antibodies were used, sections were incubated with mouse IgG blocking reagent (Vector Laboratories, Burlingame, CA, USA). The sections were then stained with the following primary antibodies: rabbit anti-Olig-2 (1:500, AB9610; Sigma-Aldrich, St. Louis, MO, USA), goat anti-platelet-derived growth factor receptor α (PDGFRα; 1:200, AF1062; R&D Systems, Minneapolis, MN, USA), mouse anti-CC1 (1:100, OP80; Sigma-Aldrich), and rat anti-myelin basic protein (MBP; 1:250, ab7349; Abcam, Cambridge, UK). The following secondary antibodies (1:500; Thermo Fisher Scientific) were used: Alexa Fluor 350-conjugated donkey anti-goat, Alexa Fluor 488-conjugated donkey anti-rabbit, Alexa Fluor 594-conjugated donkey anti-mouse, Alexa Fluor 594-conjugated goat anti-rat, and Alexa Fluor 594-conjugated goat anti-rabbit. The immunostained sections were observed under a fluorescence microscope (BZ9000; Keyence, Osaka, Japan).

### 2.8. Western Blotting Analysis

Spinal cord white matter tissues from 10-week-old and 12-month-old control and KO mice were homogenized in T-PER (Thermo Fisher Scientific) containing protease inhibitor cocktail set I (Fujifilm Wako Pure Chemicals, Osaka, Japan) and centrifuged at 10,000× *g* for 5 min. Proteins in the supernatant were separated using 10% SDS–polyacrylamide gel electrophoresis and electrophoretically transferred to polyvinylidene difluoride membranes. Membranes were blocked for 30 min at room temperature with 5% skimmed milk (Fujifilm Wako Pure Chemical) and incubated with the following primary antibodies: rabbit anti-MBP (1:1000, ab40390; Abcam), rabbit anti-PLP (1:1000, ab105784; Abcam), rabbit anti-phosphatase and tensin homolog deleted on chromosome 10 (PTEN; 1:500, ab32199; Abcam), rabbit anti-lamin B1 (1:1000, ab16048; Abcam), rabbit anti-leucine zipper like transcription regulator 1 (LZTR1; 1:1000, ab106655; Abcam), rabbit anti-R-RAS (1:500, ab154962; Abcam), rabbit anti-protein kinase B (AKT; 1:1000, #9272; Cell Signaling Technology, Danvers, MA, USA), rabbit anti-phospho-AKT (p-AKT; 1:2000, #4060; Cell Signaling Technology), rabbit anti-extracellular signal-regulated kinase 1/2 (ERK1/2; 1:1000, #9102; Cell Signaling Technology), and rabbit anti-phospho-ERK1/2 (p-ERK1/2; 1:1000, #9101; Cell Signaling Technology), followed by the secondary horseradish-peroxidase-conjugated anti-rabbit IgG antibody. Peroxide-conjugated mouse anti-glyceraldehyde-3-phosphate dehydrogenase (GAPDH; 1:5000, 015-25473; Fujifilm Wako Pure Chemical) was also used to analyze GAPDH expression. The blots were visualized using Amersham ECL Detection Reagents (Cytiva, Marlborough, MA, USA). The expression of each protein was normalized to that of GAPDH.

### 2.9. Statistical Analysis

All measurements were expressed as mean ± standard deviation. The Mann–Whitney U-test was used to compare two groups. One-way analysis of variance (ANOVA), followed by Tukey’s post hoc test, was performed for multiple comparisons. A *p* value of 0.05 was considered statistically significant. Graphs were generated and statistical analysis was performed using Prism 10 software (GraphPad Software, Boston, MA, USA).

## 3. Results

### 3.1. Body Size and Morphology

**Figure 1:**
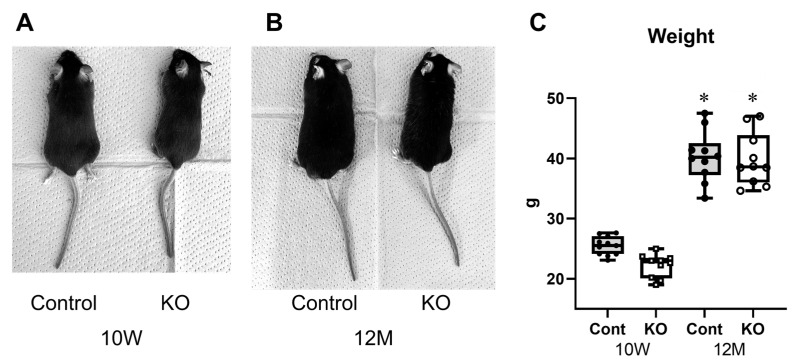
Characteristics of the myelin-specific miR-23a/b cluster knockout mice. Myelin-specific miR-23a/b cluster knockout mice (KO). (**A**) Appearance of the 10-week-old (10 W) control (Cont) and KO mice. (**B**) Appearance of the 12-month-old (12 M) Cont and KO mice. (**C**) Body weights of Cont and KO mice at 10 weeks and 12 months of age (*n* = 8 per group). * Significant difference compared to 10 W mice, *p* < 0.05.

KO mice treated with tamoxifen from seven days of age were slightly smaller than the control mice at 10 weeks of age but showed no morphological abnormalities (Figure 1A). KO mice treated with tamoxifen from 10 weeks of age were the same size as control mice at 12 months of age with no morphological abnormalities (Figure 1B). Body weights were 25.25 ± 1.45 g for 10-week-old control mice, 22.19 ± 1.93 g for 10-week-old KO mice, 40.30 ± 4.24 g for 12-month-old control mice, and 39.85 ± 4.39 g for 12-month-old KO mice (*n* = 10/group). One-way ANOVA showed significant differences between the groups (F(3,36) = 84.26, *p* < 0.01). Both control and KO mice were significantly heavier at 12 months than at 10 weeks of age (*p* < 0.01); however, there was no significant difference in body weight between the control and KO mice at 10 weeks (*p* = 0.18) or 12 months (*p* = 0.99) of age.

### 3.2. Expression of the miR-23-27-24 Cluster

**Figure 2:**
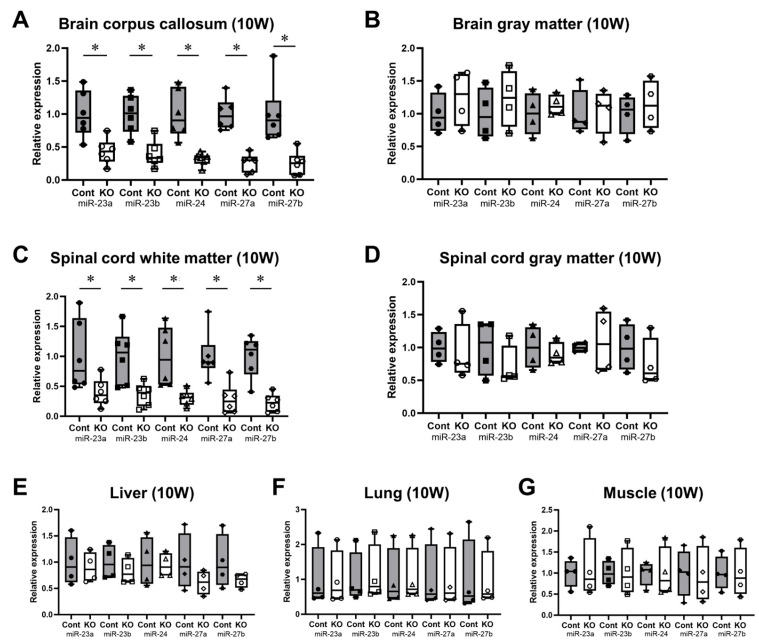
Expression levels of miRNAs in the miR-23a/b clusters (miR-23a, miR-23b, miR-24, miR-27a, and miR-27b) at 10 weeks of age. Control (Cont) and myelin-specific miR-23a/b cluster knockout mice (KO); *n* = 6 per group. (**A**) Brain corpus callosum. (**B**) Brain gray matter. (**C**) Spinal cord white matter. (**D**) Spinal cord gray matter. (**E**) Liver. (**F**) Lung. (**G**) Skeletal muscle. * Significant difference between Cont and KO mice, *p* < 0.05. 10W = 10-week-old mice.

**Figure 3:**
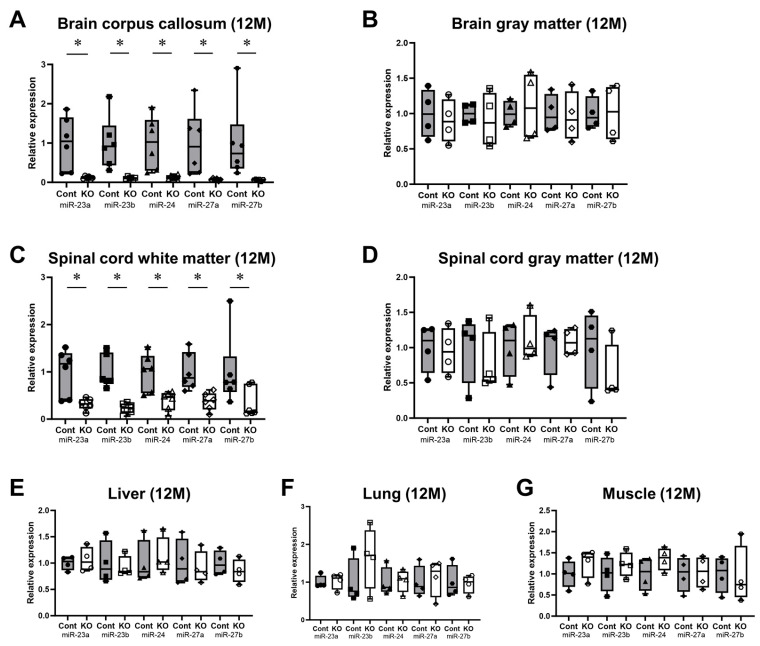
Expression levels of miRNAs in the miR-23a/b clusters (miR-23a, miR-23b, miR-24, miR-27a, and miR-27b) at 12 months of age. Control (Cont) and myelin-specific miR-23a/b cluster knockout mice (KO); *n* = 6 per group. (**A**) Brain corpus callosum. (**B**) Brain gray matter. (**C**) Spinal cord white matter. (**D**) Spinal cord gray matter. (**E**) Liver. (**F**) Lung. (**G**) Skeletal muscle. * Significant difference between Cont and KO mice, *p* < 0.05. 12 M = 12-month-old mice.

The expression levels of all miRNAs in the miR-23a/b clusters were assessed using real-time PCR (*n* = 6/group). The expression levels of these miRNAs in 10-week-old control and KO mice are shown in Table 1. The expression of all miRNAs in the corpus callosum of KO mice was significantly lower than in control mice; however, in the brain gray matter, no significant differences were observed (Figure 2A,B). Similarly, in the spinal cord, the expression of all miRNAs in the white matter was significantly lower in 10-week-old KO mice than in control mice; no significant differences were observed in the brain gray matter (Figure 2C,D). In addition, the expression of all miRNAs in the liver, lungs, and skeletal muscle was not significantly different between the KO and control mice (Figure 2E–G). The expression levels of these miRNAs in 12-month-old control and KO mice are shown in Table 2. Similarly, KO mice showed a significant white-matter-specific decrease in the expression of all miRNAs in the brain and spinal cord (Figure 3A,C), with no significant decrease observed in the gray matter of the brain or spinal cord (Figure 3B,D) nor in organs other than the CNS (Figure 3E–G). These results show that the expression of all miRNAs in the miR-23a/b clusters is specifically suppressed in the white matter of the brain and spinal cord in 10-week-old mice treated with tamoxifen from 7 days of age and in 12-month-old mice treated with tamoxifen from 10 weeks of age.

### 3.3. Impaired Motor Function in miR-23a/b Cluster-Deficient Mice

**Figure 4:**
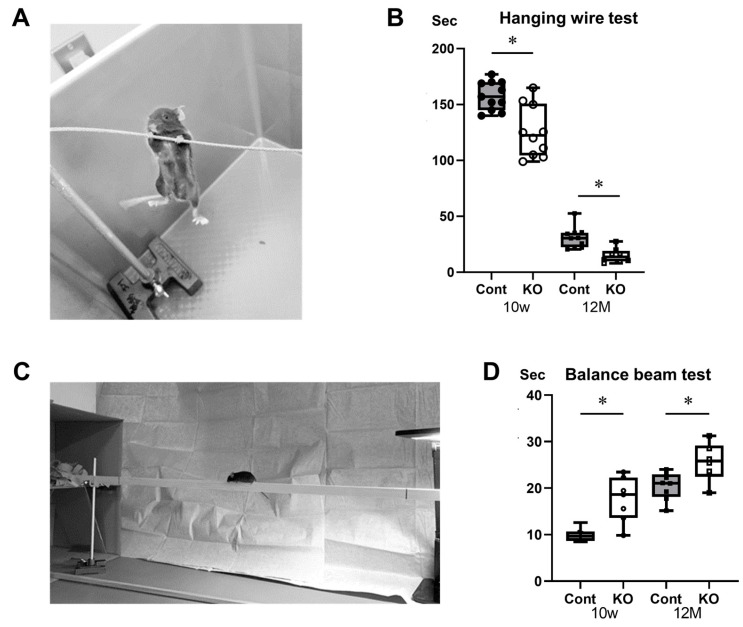
Behavioral assessments. Control (Cont) and myelin-specific miR-23a/b cluster knockout mice (KO); *n* = 8 per group. (**A**) Photograph of the hanging wire test. (**B**) Number of seconds from the start of the test to the time the mice fell during the hanging wire test. * Significant difference between Cont and KO mice, *p* < 0.05. (**C**) Photograph of the balance beam test. (**D**) Number of seconds for the mice to cross the beam in the balance beam test. * Significant difference between Cont and KO mice, *p* < 0.05.

Hanging wire and balance beam tests were used to assess the effects of miR-23a/b cluster defects on locomotor function in mice (Figure 4A,C). In the hanging wire test, the time until the mice fell was 157.91 ± 12.72 s for 10-week-old control mice, 125.68 ± 23.10 s for 10-week-old KO mice, 30.95 ± 9.49 s for 12-month-old control mice, and 14.97 ± 6.55 s for 12-month-old KO mice (*n* = 8/group). KO mice fell significantly faster than control mice at 10 weeks and 12 months of age (*p* < 0.01; Figure 4B). The time required for mice to cross the beam in the balance beam test was 9.88 ± 1.39 s for 10-week-old control mice, 17.55 ± 4.85 s for 10-week-old KO mice, 20.46 ± 2.95 s for 12-month-old control mice, and 25.67 ± 4.20 s for 12-month-old KO mice (*n* = 8/group). The KO mice took significantly longer to cross the beam than the control mice at 10 weeks (*p* < 0.01) and 12 months (*p* = 0.03) of age (Figure 4D). These results suggest that KO mice have reduced locomotor function compared with control mice at 10 weeks and 12 months of age.

### 3.4. Hypoplasia of CNS Myelin Due to Deficiency of miR-23a/b Clusters

**Figure 5:**
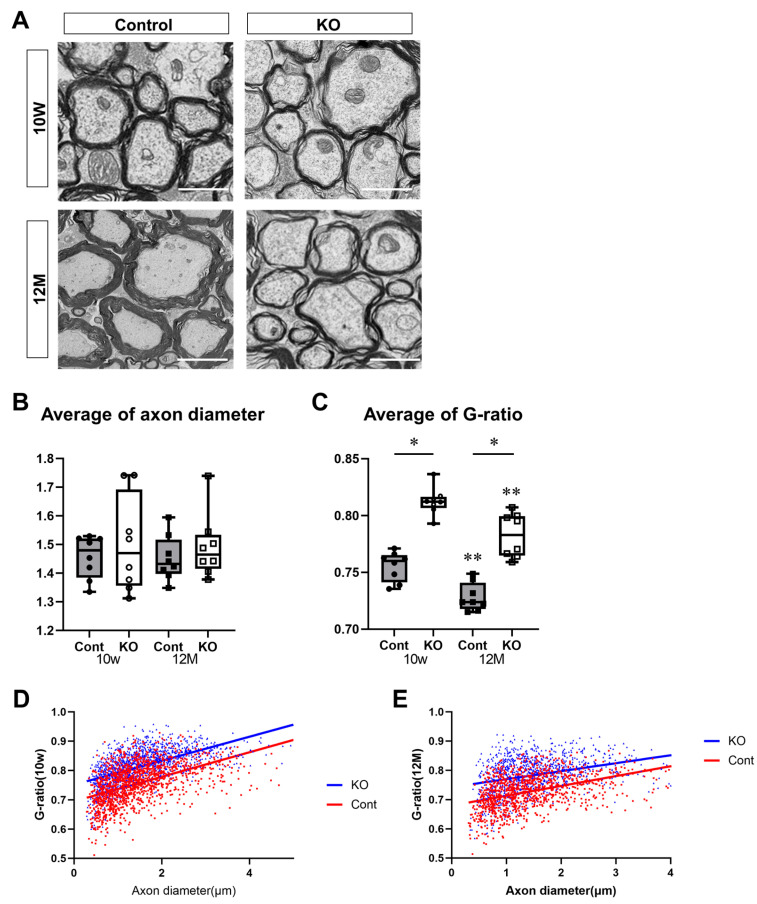
Evaluation of the myelin sheath via electron microscopy. Control (Cont) and myelin-specific miR-23a/b cluster knockout mice (KO). We evaluated 120–180 axons per mouse (*n* = 8 per group). (**A**) Electron micrographs of the spinal cord cross-sections of Cont and KO mice at 10 weeks (10 W) and 12 months (12 M) of age. Scale bar: 2 μm. (**B**) Average axon diameter in Cont and KO mice at 10 W and 12 M of age. (**C**) Average G-ratio in Cont and KO mice at 10 W and 12 M of age. The G-ratio is the ratio of the diameter of the axon without a myelin sheath to the diameter of the axon with a myelin sheath. * Significant difference between Cont and KO mice, ** significantly lower than 10 W mice, *p* < 0.05. (**D**,**E**) Scatter plots of G-ratios versus axon diameter for Cont (red) and KO (blue) mice at (**D**) 10 W and (**E**) 12 M of age.

To determine the effect of miR-23a/b cluster deletion on myelination in the spinal cord, the thickness of the myelin sheath was assessed using electron microscopy. KO mice had thinner myelin sheaths than control mice at 10 weeks and 12 months of age (Figure 5A). The thickness of the myelin sheath was evaluated using the G-ratio. The diameter of axons without a myelin sheath was 1.46 ± 0.07 µm for control mice and 1.50 ± 0.17 µm for KO mice at 10 weeks of age and 1.45 ± 0.08 µm for control mice and 1.49 ± 0.11 µm for KO mice at 12 months of age (*n* = 8/group). One-way ANOVA showed no significant differences between the groups (F(3,28) = 0.37, *p* = 0.78). There were no significant differences observed between 10-week-old and 12-month-old mice nor between control and KO mice (Figure 5B). The G-ratios were 0.76 ± 0.01 for 10-week-old control mice, 0.81 ± 0.01 for 10-week-old KO mice, 0.73 ± 0.01 for 12-month-old control mice, and 0.78 ± 0.02 for 12-month-old KO mice (*n* = 8/group). One-way ANOVA showed significant differences between the groups (F(3,28) = 49.81, *p* < 0.01). The G-ratio was significantly higher in KO mice than in the control mice at 10 weeks and 12 months of age (*p* < 0.01). In addition, the G-ratio was significantly lower at 12 months than at 10 weeks of age in the control and KO mice (*p* < 0.01; Figure 5C). The G-ratios in KO mice were higher than in control mice at 10 weeks and 12 months of age, regardless of the axon diameter (Figure 5D,E).

**Figure 6:**
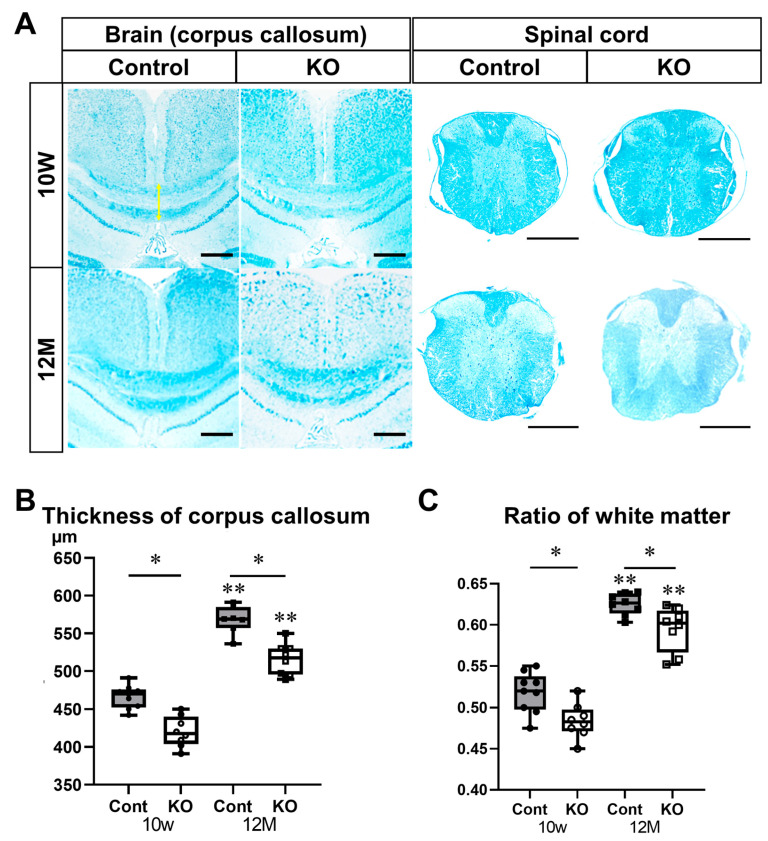
Luxol Fast Blue staining of brain and spinal cord sections. Control (Cont) and myelin-specific miR-23a/b cluster knockout mice (KO); *n* = 8 per group. (**A**) Luxol Fast Blue stained images of the coronal surface of the corpus callosum and transverse section of the thoracic spinal cord of 10-week-old (10 W) and 12-month-old (12 M) Cont and KO mice. The yellow arrow indicates the thickness of the corpus callosum. Scale bar: 500 μm. (**B**) Thickness of the corpus callosum (µm). * Significant difference between Cont and KO mice, ** significantly thicker than 10 W mice, *p* < 0.05. (**C**) Ratio of the white matter area to the total area of the spinal cord (ratio of white matter). * Significant difference between Cont and KO mice, ** significantly higher than 10 W mice, *p* < 0.05.

To further evaluate the myelin sheath, white matter was assessed using Luxol Fast Blue staining of the brain and spinal cord (Figure 6). The thicknesses of the brain corpus callosum were 466.22 ± 15.32 µm in 10-week-old control mice, 420.13 ± 20.22 µm in 10-week-old KO mice, 568.00 ± 18.11 µm in 12-month-old control mice, and 515.75 ± 21.12 µm in 12-month-old KO mice (*n* = 8/group). The brain corpus callosum was significantly thinner in KO mice than in the control mice at 10 weeks and 12 months of age (*p* < 0.01). In addition, the brain corpus callosum was thicker at 12 months than at 10 weeks of age in the control and KO mice (*p* < 0.01; Figure 6A,B). In transverse sections of the spinal cord, the ratios of white matter to spinal cord cross-sectional area were 0.52 ± 0.02 in 10-week-old control mice, 0.48 ± 0.02 in 10-week-old KO mice, 0.63 ± 0.01 in 12-month-old control mice, and 0.60 ± 0.03 in 12-month-old KO mice (*n* = 8/group). The ratio of white matter to spinal cord cross-sectional area was significantly lower in KO mice than in control mice at 10 weeks (*p* = 0.02) and 12 months (*p* = 0.04) of age. In addition, the ratio of white matter to spinal cord cross-sectional area was significantly higher at 12 months than at 10 weeks of age in the control and KO mice (*p* < 0.01; Figure 6A,C).

These electron microscopy and Luxol Fast Blue staining findings indicate that myelin sheaths continue to develop with age until 12 months and that myelin-specific deletion of miR-23a/b clusters from 7 days to 10 weeks of age and from 10 weeks to 12 months of age results in hypoplasia of myelin in the CNS.

### 3.5. Effects of miR-23a/b Cluster Deletion on Oligodendrocyte Differentiation and Myelination

**Figure 7:**
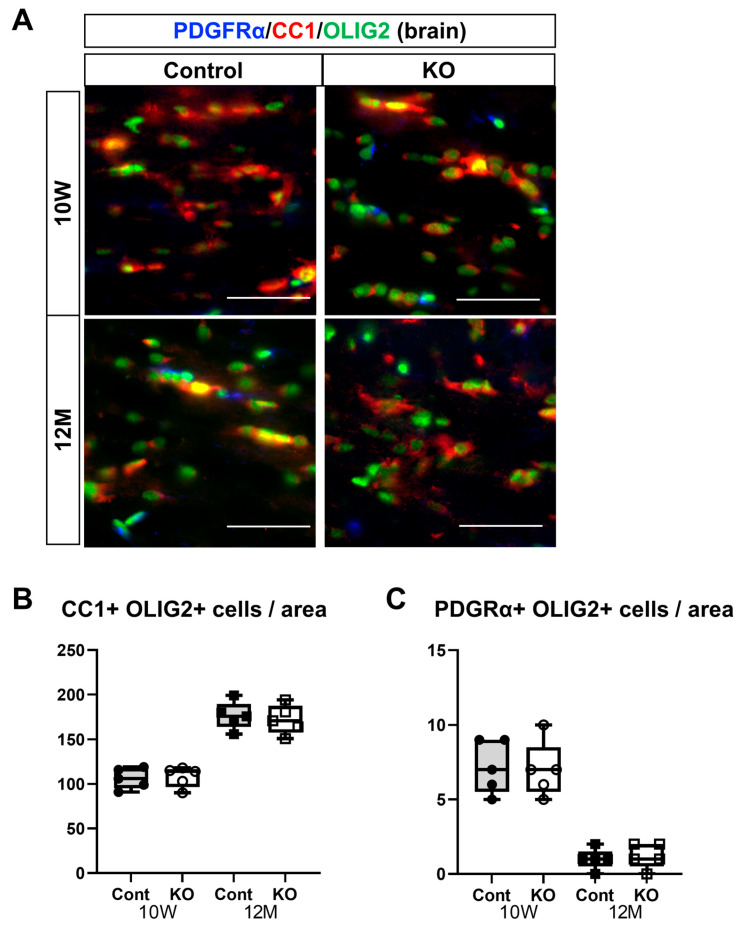
Immunostaining of oligodendrocytes and their progenitor cells in brain sections. Control (Cont) and myelin-specific miR-23a/b cluster knockout mice (KO); *n* = 7 per group. (**A**) Immunostaining images of the brain corpus callosum stained with platelet-derived growth factor α (PDGFα), CC1, and OLIG2 of 10-week-old (10 W) and 12-month-old (12 M) Cont and KO mice. (**B**) Number per unit area of mature oligodendrocytes showing double-positivity for CC1 and OLIG2. (**C**) Number per unit area of oligodendrocyte progenitors showing double-positivity for PDGFRα and OLIG2.

**Figure 8:**
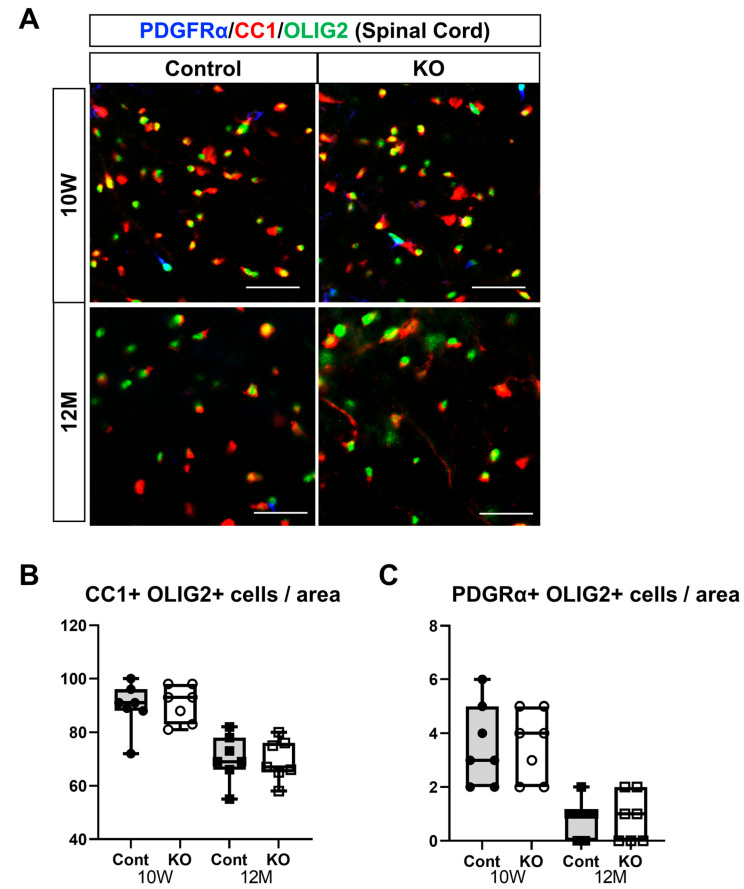
Immunostaining of oligodendrocytes and their progenitor cells in brain sections. Control (Cont) and myelin-specific miR-23a/b cluster knockout mice (KO); *n* = 7 per group. (**A**) Immunostaining images of the spinal cord white matter stained with platelet-derived growth factor α (PDGFα), CC1, and OLIG2 of 10-week-old (10 W) and 12-month-old (12 M) Cont and KO mice. (**B**) Number per unit area of mature oligodendrocytes showing double-positivity for CC1 and OLIG2. (**C**) Number per unit area of oligodendrocyte progenitors showing double-positivity for PDGFRα and OLIG2.

To assess the effects of the myelin-specific deletion of miR-23a/b clusters on oligodendrocyte differentiation and maturation, immunostaining of mature oligodendrocytes and OPCs was performed on brain corpus callosum and spinal cord white matter sections. Mature oligodendrocytes were identified as double-positive for CC1 and OLIG2, whereas OPCs were double-positive for PDGFRα and OLIG2 (Figure 7A and Figure 8A). In the corpus callosum, the number of mature oligodendrocytes (double-positive for CC1 and OLIG2) per unit area was 106.20 ± 11.65 in 10-week-old control mice, 108.00 ± 11.55 in 10-week-old KO mice, 176.60 ± 15.49 in 12-month-old control mice, and 172.20 ± 16.36 in 12-month-old KO mice (*n* = 5/group). One-way ANOVA showed significant differences between the groups (F(3,16) = 38.97, *p* < 0.01). The number of mature oligodendrocytes at 12 months of age was significantly greater than at 10 weeks of age in the control and KO mice (*p* < 0.01). However, no significant difference was observed between the control and KO mice at 10 weeks (*p* = 1.00) or 12 months (*p* = 0.96) of age (Figure 7B). 

The number of OPCs (double-positive for PDGFRα and OLIG2) per unit area in the corpus callosum was 7.20 ± 1.79 in 10-week-old control mice, 7.00 ± 1.87 in 10-week-old KO mice, 1.00 ± 0.71 in 12-month-old control mice, and 1.20 ± 0.84 in 12-month-old KO mice (*n* = 5/group). One-way ANOVA showed significant differences between the groups (F(3,16) = 30.41, *p* < 0.01). The number of OPCs at 12 months of age was significantly greater than at 10 weeks of age in the control and KO mice (*p* < 0.01). However, no significant difference was observed between the control and KO mice at 10 weeks or 12 months of age (*p* = 1.00; Figure 7C). 

In the spinal cord, the number of mature oligodendrocytes per unit area was 89.57 ± 8.81 in 10-week-old control mice, 90.57 ± 6.80 in 10-week-old KO mice, 70.29 ± 8.75 in 12-month-old control mice, and 69.57 ± 7.68 in 12-month-old KO mice (*n* = 7/group). One-way ANOVA showed significant differences between the groups (F(3,24) = 14.62, *p* < 0.01). The number of mature oligodendrocytes at 12 months was significantly greater than at 10 weeks of age in the control and KO mice (*p* < 0.01). However, no significant difference was observed between the control and KO mice at 10 weeks or 12 months of age (*p* = 1.00; (Figure 8B). The number of OPCs per unit area in the spinal cord was 3.57 ± 1.51 in 10-week-old control mice, 3.86 ± 1.77 in 10-week-old KO mice, 0.86 ± 0.69 in 12-month-old control mice, and 0.86 ± 0.90 in 12-month-old KO mice (*n* = 7/group). One-way ANOVA showed significant differences between the groups (F(3,24) = 11.40, *p* < 0.01). The number of OPCs at 12 months was significantly greater than that at 10 weeks of age in the control and KO mice (*p* < 0.01). However, no significant difference was observed between the control and KO mice at 10 weeks (*p* = 0.98) or 12 months (*p* = 1.00) of age (Figure 8C).

**Figure 9:**
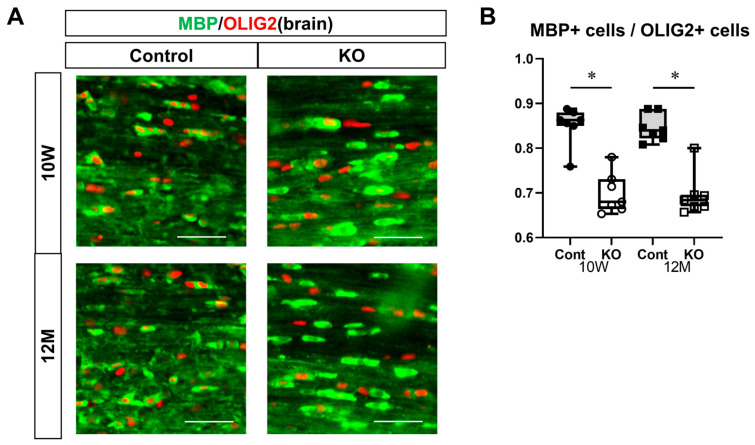
Immunostaining of myelinating oligodendrocytes in brain sections. Control (Cont) and myelin-specific miR-23a/b cluster knockout mice (KO); *n* = 7 per group. (**A**) Immunostaining images of the brain corpus callosum stained with myelin basic protein (MBP) and OLIG2 of 10-week-old (10 W) and 12-month-old (12 M) Cont and KO mice. (**B**) Ratio of the number of myelinating oligodendrocytes showing double-positivity for MBP and OLIG2 to the number of OLIG2-positive oligodendrocytes. * Significant difference between Cont and KO mice, *p* < 0.05.

To evaluate myelinating oligodendrocytes, sections of the corpus callosum from 10-week-old and 12-month-old mice were immunostained for MBP and OLIG2 (Figure 9A). The ratio of myelinating oligodendrocytes (double-positive for MBP and OLIG2) to total OLIG2-positive cells was calculated. The ratio of myelinating oligodendrocytes was 0.85 ± 0.04 in 10-week-old control mice, 0.70 ± 0.04 in 10-week-old KO mice, 0.85 ± 0.03 in 12-month-old control mice, and 0.70 ± 0.05 in 12-month-old KO mice (*n* = 7/group). One-way ANOVA showed significant differences between the groups (F(3,24) = 29.99, *p* < 0.01). The ratio of myelinating oligodendrocytes in KO mice (0.70 ± 0.04) was significantly lower than in the control mice at 10 weeks and 12 months of age (*p* < 0.01). However, no significant difference was observed between 10-week-old and 12-month-old control and KO mice (*p* = 1.00; Figure 9B).

These immunostaining results suggest that the myelin-specific deletion of miR-23a/b clusters does not affect the differentiation of OPCs into mature oligodendrocytes but decreases the number of myelinating oligodendrocytes.

### 3.6. Decreased Expression of MBP and PLP Due to the Deletion of miR-23a/b Clusters

**Figure 10:**
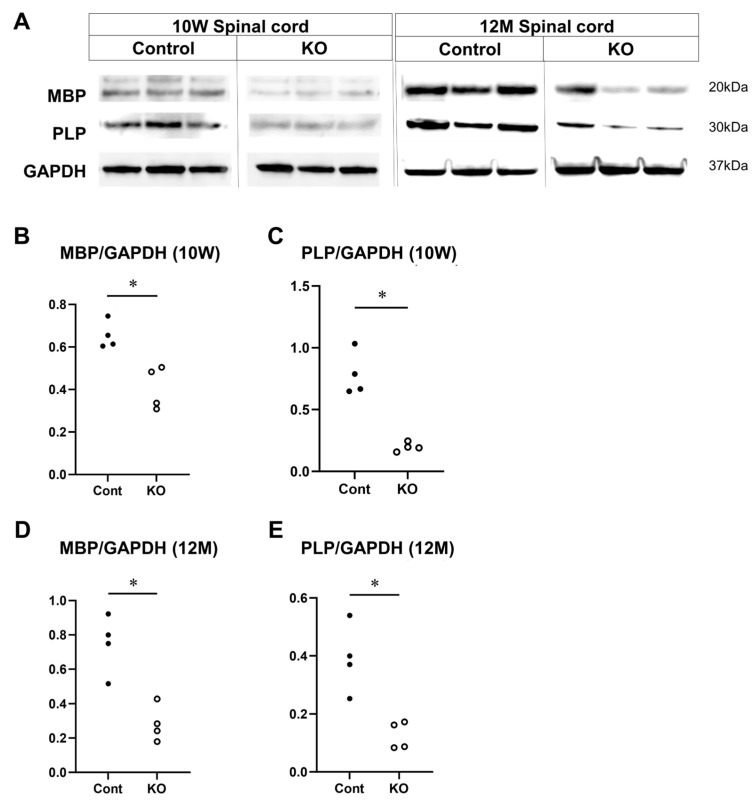
Expression of MBP and PLP in the spinal cord. Control (Cont) and myelin-specific miR-23a/b cluster knockout mice (KO); *n* = 4 per group. (**A**) Representative Western blots of myelin basic protein (MBP), proteolipid protein (PLP), and glyceraldehyde-3-phosphate dehydrogenase (GAPDH) of 10-week-old (10 W) and 12-month-old (12 M) Cont and KO mice. (**B**) Relative expression of MBP to GAPDH at 10 weeks of age. (**C**) Relative expression of PLP to GAPDH at 10 weeks of age. (**D**) Relative expression of MBP to GAPDH at 12 months of age. (**E**) Relative expression of PLP to GAPDH at 12 months of age. * Significant difference between Cont and KO mice in all test groups, *p* < 0.05.

To determine the effects of the myelin-specific miR-23a/b cluster deletion on MBP and PLP—proteins important for myelin formation—their expression levels were measured in the spinal cord white matter at 10 weeks and 12 months of age via Western blotting (*n* = 4/group; Figure 10A). The relative expression level of MBP to GAPDH at 10 weeks of age was 0.65 ± 0.06 in control mice and 0.42 ± 0.11 in KO mice (*p* = 0.03; Figure 10B). The relative expression level of PLP to GAPDH was 0.78 ± 0.18 in control mice and 0.20 ± 0.04 in KO mice (*p* = 0.03; Figure 10C). At 12 months of age, the relative expression level of MBP to GAPDH was 0.75 ± 0.17 in control mice and 0.28 ± 0.11 in KO mice (*p* = 0.03; Figure 10D). The relative expression level of PLP to GAPDH was 0.39 ± 0.12 in control mice and 0.13 ± 0.05 in KO mice (*p* = 0.03; Figure 10E). These results show that the myelin-specific deletion of miR-23a/b clusters reduces MBP and PLP expression in spinal cord white matter.

### 3.7. Candidate Target Molecules for miR-23a/b Clusters

**Figure 11:**
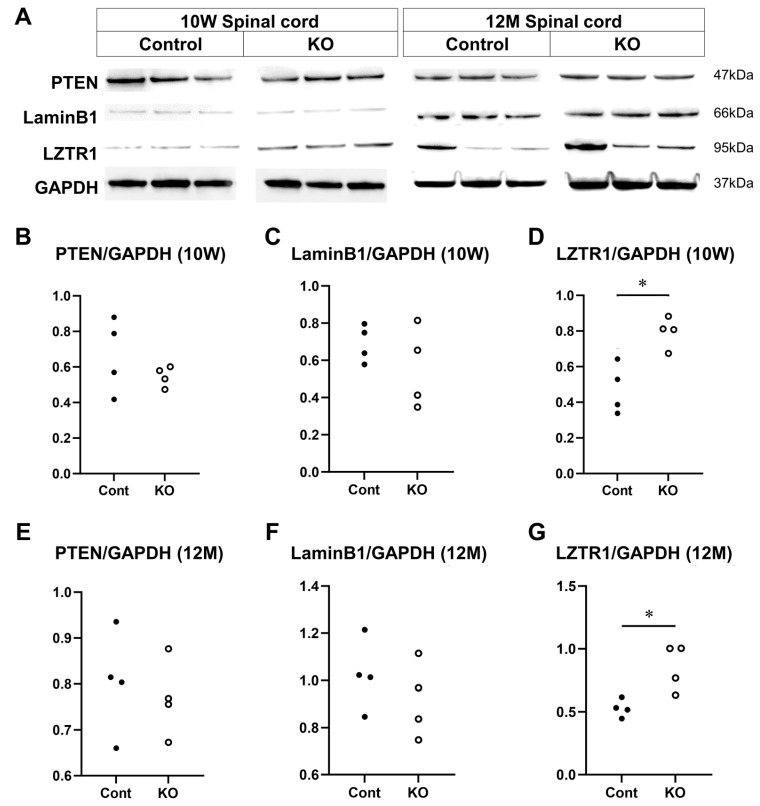
Expression of candidate targets of miR-23a/b clusters. Control (Cont) and myelin-specific miR-23a/b cluster knockout mice (KO); *n* = 4 per group. (**A**) Representative Western blots of phosphatase and tensin homolog deleted on chromosome 10 (PTEN), lamin B1, LZTR1, and glyceraldehyde-3-phosphate dehydrogenase (GAPDH) of 10-week-old (10 W) and 12-month-old (12 M) Cont and KO mice. (**B**) Relative expression of PTEN to GAPDH at 10 weeks of age. (**C**) Relative expression of lamin B1 to GAPDH at 10 weeks of age. (**D**) Relative expression of LZTR1 to GAPDH at 10 weeks of age. (**E**) Relative expression of PTEN to GAPDH at 12 months of age. (**F**) Relative expression of lamin B1 to GAPDH at 12 months of age. (**G**) Relative expression of LZTR1 to GAPDH at 12 months of age. * Significant difference between Cont and KO mice in all test groups, *p* < 0.05.

The expression of PTEN, lamin B1, and LZTR1, which have been reported as candidate miRNA target molecules for miR-23a/b clusters in myelin, was evaluated using Western blotting of the spinal cord white matter at 10 weeks and 12 months of age (*n* = 4/group) (Figure 11A). At 10 weeks of age, the relative expression level of PTEN to GAPDH was 0.66 ± 0.21 in control mice and 0.55 ± 0.06 in KO mice (*p* = 0.69; Figure 11B). The relative expression level of lamin B1 to GAPDH was 0.69 ± 0.10 in control mice and 0.56 ± 0.22 in KO mice (*p* = 0.56; Figure 11C). The relative expression level of LZTR1 to GAPDH was 0.47 ± 0.14 in control mice and 0.79 ± 0.09 in KO mice (*p* = 0.03; Figure 11D). At 12 months of age, the relative expression level of PTEN to GAPDH was 0.81 ± 0.11 in control mice and 0.83 ± 0.15 in KO mice (*p* = 0.89; Figure 11E). The relative expression level of lamin B1 to GAPDH was 1.09 ± 0.09 in control mice and 0.92 ± 0.17 in KO mice (*p* = 0.20; Figure 11F). The relative expression level of LZTR1 to GAPDH was 0.53 ± 0.07 in control mice and 0.85 ± 0.18 in KO mice (*p* = 0.03; Figure 11G). These results indicate that the myelin-specific deletion of miR-23a/b clusters does not alter PTEN or lamin B1 expression but increases that of LZTR1 at 10 weeks and 12 months of age.

### 3.8. Signaling Factors That are Altered by miR-23a/b Cluster Deletion

**Figure 12:**
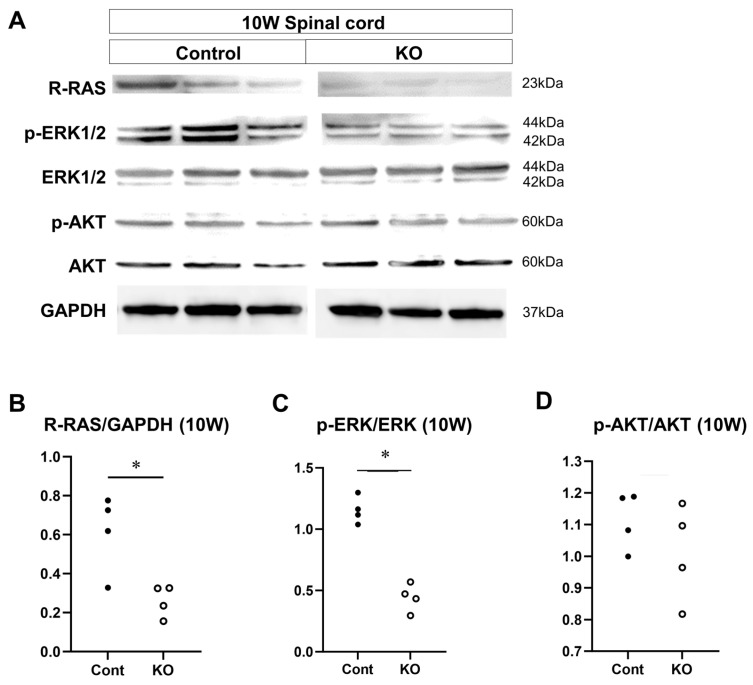
Expression of signaling factors at 10 weeks of age. Control (Cont) and myelin-specific miR-23a/b cluster knockout mice (KO); *n* = 4 per group. (**A**) Representative Western blots of R-RAS, phospho-ERK1/2 (p-ERK1/2), ERK1/2, phospho-AKT (p-AKT), AKT, and glyceraldehyde-3-phosphate dehydrogenase (GAPDH) in the spinal cord white matter of 10-week-old (10 W) Cont and KO mice. (**B**) Relative expression of R-RAS to GAPDH. (**C**) Relative expression of p-ERK1/2 to ERK1/2. * Significant difference between Cont and KO mice, *p* < 0.05. (**D**) Relative expression of p-AKT to AKT.

**Figure 13:**
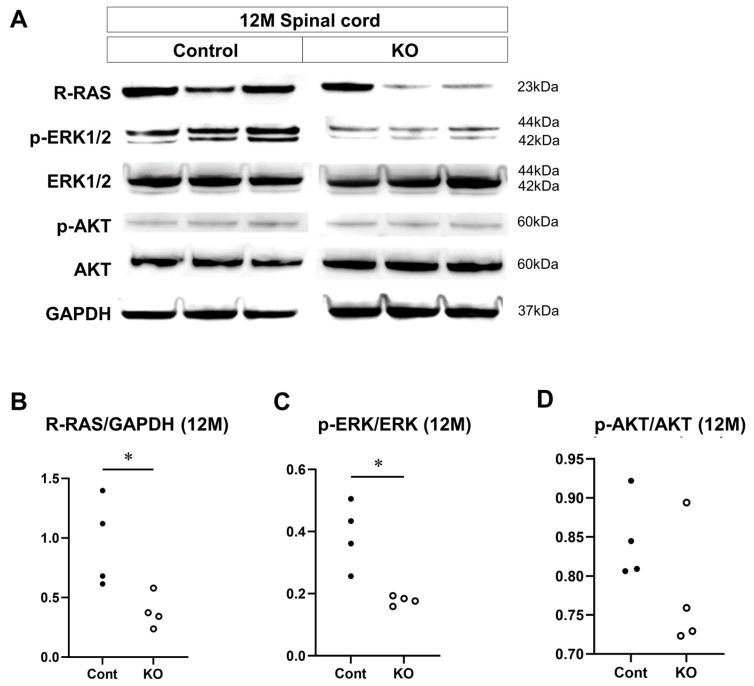
Expression of signaling factors at 12 months of age. Control (Cont) and myelin-specific miR-23a/b cluster knockout mice (KO); *n* = 4 per group. (**A**) Representative Western blots of R-RAS, phospho-ERK1/2 (p-ERK1/2), ERK1/2, phospho-AKT (p-AKT), AKT, and glyceraldehyde-3-phosphate dehydrogenase (GAPDH) in the spinal cord white matter of 12-month-old (12 M) Cont and KO mice. (**B**) Relative expression of R-RAS to GAPDH. (**C**) Relative expression of p-ERK1/2 to ERK1/2. * Significant difference between Cont and KO mice, *p* < 0.05. (**D**) Relative expression of p-AKT to AKT.

The effects of the deletion of myelin-specific miR-23a/b clusters on the expression of signaling factors involved in myelin formation were evaluated through Western blotting of the spinal cord white matter at 10 weeks and 12 months of age (*n* = 4/group; Figure 12A and Figure 13A). At 10 weeks of age, the relative expression level of R-RAS to GAPDH was 0.61 ± 0.20 in control mice and 0.26 ± 0.08 in KO mice (*p* = 0.03; Figure 12B). The ratio of p-ERK1/2 to total ERK1/2 was 1.15 ± 0.11 in control mice and 0.44 ± 0.11 in KO mice (*p* = 0.03; Figure 12C). In contrast, the ratio of p-AKT to total AKT was 1.11 ± 0.09 in control mice and 1.01 ± 0.15 in KO mice (*p* = 0.34; Figure 12D). At 12 months of age, the relative expression level of R-RAS to GAPDH was 0.95 ± 0.37 in control mice and 0.38 ± 0.14 in KO mice (*p* = 0.03; Figure 13B). The ratio of p-ERK1/2 to total ERK1/2 was 0.39 ± 0.11 in control mice and 0.18 ± 0.01 in KO mice (*p* = 0.03; Figure 13C). In contrast, the ratio of p-AKT to total AKT was 1.06 ± 0.17 in control mice and 0.89 ± 0.15 in KO mice (*p* = 0.20; Figure 13D). These results indicate that the deletion of myelin-specific miR-23a/b clusters reduced R-RAS expression and ERK phosphorylation, but not AKT phosphorylation, at 10 weeks and 12 months of age.

## 4. Discussion

This study demonstrated that the myelin-specific deletion of miR-23a/b clusters resulted in suppressed myelination and impaired motor function. This deletion also suppressed the expression of MBP and PLP, which are both critical for myelin formation, as well as the expression of R-RAS and the phosphorylation of ERK, known to promote myelination. In addition, this targeted deletion upregulated LZTR1, a potential target molecule of the miR-23a/b clusters. These observations were consistent in 10-week-old KO mice treated with tamoxifen from 7 days of age and in 12-month-old KO mice treated from 10 weeks of age.

Previous studies have reported the expression of miR-23a/b clusters in the liver, lungs, skeletal muscles, and CNS [14,18,19]. In this context, the present study assessed changes in the expression of these clusters in the organs of myelin-specific miR-23a/b cluster-deficient mice. In these mice, the expression of miR-23a/b clusters was reduced solely in the white matter of the brain and spinal cord, suggesting the successful targeted deletion of miR-23a/b clusters in myelin, where the *Plp* gene is expressed.

In our previous study using mice systemically deficient in miR-23a/b clusters, differences were noted between KO and control mice in the hanging wire test but not in the balance beam test [14]. The balance beam test described in a previous study used a 10 mm wide and 50 cm long beam. However, in the present study, a narrower and longer beam (6 mm wide, 80 cm long) revealed significant differences between the KO and control mice under more challenging conditions.

We observed hypoplastic white matter and hypomyelination in KO mice, potentially attributable to aberrant oligodendrocyte differentiation. The differentiation process from OPCs to premyelinating oligodendrocytes and then to myelinating oligodendrocytes is essential for normal myelin formation [1,27,28,29,30]. Various miRNAs have been reported to regulate oligodendrocyte differentiation [31]. Transgenic mice overexpressing miR-23a, which constitutes the miR-23a/b cluster, showed increased expression of *Plp*, *Mbp*, myelin-related glycoproteins, myelin oligodendrocyte glycoproteins, and increased protein abundance of PLP and MBP, implying that miR-23a promotes differentiation into myelinating oligodendrocytes [13,32]. However, the effects of miR-23a downregulation on oligodendrocyte differentiation and myelination have not yet been elucidated. In contrast, the inhibitory effect of miR-27a, another component of miR-23a/b clusters, on myelination has been reported [12]. In vitro studies have shown that the downregulation of miR-27a reduces NG2 expression, a marker for OPCs, whereas its overexpression increases NG2 levels, leading to cell cycle arrest in OPCs. In addition, in vitro experiments using artificial microfibers and P4 mouse brain slices showed that the overexpression of miR-27a suppressed myelination. These findings indicate that miR-27a plays a suppressive role in OPC differentiation through cell cycle arrest and that its overexpression inhibits myelination in vitro. Intriguingly, both suppression and overexpression of miR-27a in vitro reduced MBP expression, indicating the importance of maintaining steady-state miR-27a levels for differentiation into myelinating oligodendrocytes and that miR-27a suppression does not necessarily promote myelination. Although there are no previous studies showing the effect of the downregulation of either miR-23a or miR-27a on myelination in vivo, our previous studies and the present study show that the deletion of miR-23a/b clusters containing these miRNAs suppresses myelination in vivo [14]. 

The *Plp* gene is expressed not only in myelinating oligodendrocytes expressing the PLP protein but also in OPCs [33]. A previous study using *Plp-Cre^ERT^*;ROSA26-LacZ double-transgenic mice reported β-gal activity in NG2-positive OPCs [34]. However, in the present study, no difference was found in the number of OPCs or mature oligodendrocytes between KO mice and controls, indicating that miR-23a/b cluster deletion did not affect OPC differentiation. Nevertheless, KO mice showed decreased MBP and PLP expression and fewer myelinating oligodendrocytes expressing MBP. These findings suggest that miR-23a/b clusters are more important for myelin formation in mature oligodendrocytes than for the differentiation of OPCs.

In our previous study, mice with a systemic deficiency in miR-23a/b clusters exhibited myelin hypoplasia compared to controls at 4 and 10 weeks and 12 months of age [14]. However, the impact of miR-23a/b cluster deficiency on myelination during postnatal growth and subsequent aging phases remains uncertain, as miR-23a/b clusters are defective at the embryonic stage. Therefore, inducible miR-23a/b cluster KO mice were used in the present study. Evaluations were performed at 10 weeks of age in mice that started receiving tamoxifen at 7 days of age and at 12 months of age in mice that started receiving tamoxifen at 10 weeks of age. This approach enabled us to investigate the role of miR-23a/b clusters in myelination at different postnatal stages. Observations of diminished MBP and PLP expression, alongside myelin hypoplasia in KO mice at both age points, underscore the significant role of miR-23a/b clusters in myelination during postnatal growth and aging.

Lamin B1 and PTEN have been identified as target genes of miR-23a in oligodendrocytes, where miR-23a exerts a negative regulatory effect on them [13,26,35]. Overexpression of lamin B1 reduces MBP and PLP expression and branching in mature oligodendrocytes in vitro and causes abnormal myelin formation, axonal degeneration, and demyelination in vivo [26,35]. PTEN inhibits myelination by antagonizing the activity of phosphatidylinositol-3 kinase, thereby suppressing AKT activation [13,35].

Nonetheless, in our studies involving systemic and myelin-specific miR-23a/b cluster KO mice, no increase in the expression of lamin B1 or PTEN was observed, which could be attributed to the deletion of the miR-23a/b clusters. In contrast, LZTR1 was identified as a potential target molecule for the miR-23a/b clusters [14]. However, due to the complexity of the miRNA complementary interactions, ascertaining which miRNAs in a cluster contribute to LZTR1 regulation is challenging. Nevertheless, based on the nucleotide sequencing data obtained from the TargetScan database, miR-23b and miR-27a may regulate LZTR1. In addition, R-RAS—a downstream factor of LZTR1—was downregulated in KO mice. R-RAS facilitates myelination through activation of the AKT and ERK1/2 signaling pathways [36,37]. In myelin-specific miR-23a/b cluster KO mice, AKT phosphorylation was not reduced, and ERK1/2 phosphorylation was inhibited. AKT, not ERK1/2, reportedly plays an important role in promoting oligodendrocyte differentiation and the timely initiation of myelination via mechanistic target of rapamycin complex 1 (mTORC1) signaling. In contrast, ERK1/2 is primarily involved in maintaining myelin axon integrity in adulthood, largely independent of mTORC1 [38]. Therefore, the finding that ERK phosphorylation, not AKT phosphorylation, was suppressed in myelin-specific miR-23a/b cluster KO mice is consistent with the findings that the induction of adult miR-23a/b cluster deletion resulted in hypoplastic myelin with no change in OPC differentiation.

This study has certain limitations. While miR-27a is known to stimulate the Wnt/β-catenin signaling pathway in OPCs, this pathway was not explored in our study, primarily due to the absence of noticeable effects on OPC differentiation in the miR-23a/b cluster KO mice. Additionally, although PLP is expressed in Schwann cells and oligodendrocytes, the assessment of myelin in peripheral nerves was not included in this study. It is conceivable that alterations in the myelination of peripheral nerves might contribute to the observed impaired motor function in myelin-specific miR-23a/b cluster KO mice. This study identified changes in R-RAS expression and ERK1/2 phosphorylation following deletion of myelin-specific miR-23a/b clusters. However, a direct causal relationship between these factors has not been established. Several markers of myelinating oligodendrocytes have been reported; hence, other markers may be more suitable for immunohistochemical staining than MBP [39]. However, MBP was selected in this study because it is the most commonly reported marker for myelinating oligodendrocytes. Moreover, the effects of tamoxifen administration on the study results are unknown. However, the effect of tamoxifen administration appeared to be very limited; indeed, our previous study, in which tamoxifen was not administered, showed similar experimental results [14]. In addition to the miRNAs in the miR-23a/b clusters, various other miRNAs have been reported as being involved in oligodendrocyte differentiation, which may influence myelination regulation [31]. However, the interactions between these miRNAs and the miR-23a/b clusters were not determined in this study. Finally, it remains unknown whether the results of this study can be directly applied and replicated in humans, warranting further investigation. Indeed, the effects of miR-23a/b cluster deletion observed in vivo cannot be detected in humans.

## 5. Conclusions

Based on this study’s findings, we determined that the deletion of myelin-specific miR-23a/b clusters in the CNS leads to reduced MBP and PLP expression, culminating in myelin hypoplasia during the postnatal growth and aging phases.

## Figures and Tables

**Table 1:** Expression of miR-23a/b clusters in 10-week-old mice. Expression of miR-23a/b cluster microRNAs in the corpus callosum, brain gray matter, white matter and gray matter of the spinal cord at 10 weeks of age. Mean ± standard deviation of expression relative to mean expression in control mice. Statistical differences between control mice and miR-23a/b cluster knockout (KO) mice were calculated.

	Brain Corpus Callosum			Brain Gray Matter		
	Control	KO	*p* Value	Control	KO	*p* Value
miR-23a	1.00 ± 0.35	0.43 ± 0.20	<0.01	1.00 ± 0.31	1.24 ± 0.42	0.34
miR-23b	1.00 ± 0.29	0.39 ± 0.20	<0.01	1.00 ± 0.39	1.23 ± 0.44	0.69
miR-24	1.00 ± 0.37	0.32 ± 0.10	<0.01	1.00 ± 0.32	1.13 ± 0.16	0.69
miR-27a	1.00 ± 0.24	0.26 ± 0.14	<0.01	1.00 ± 0.35	1.04 ± 0.34	0.89
miR-27b	1.00 ± 0.45	0.25 ± 0.18	<0.01	1.00 ± 0.30	1.14 ± 0.37	0.69
	**Spinal cord white matter**			**Spinal cord gray matter**		
	**Control**	**KO**	***p* value**	**Control**	**KO**	***p* value**
miR-23a	1.00 ± 0.59	0.40 ± 0.23	0.03	1.00 ± 0.24	0.91 ± 0.44	0.49
miR-23b	1.00 ± 0.46	0.36 ± 0.19	0.01	1.00 ± 0.43	0.71 ± 0.31	0.49
miR-24	1.00 ± 0.50	0.30 ± 0.13	<0.01	1.00 ± 0.32	0.90 ± 0.17	0.69
miR-27a	1.00 ± 0.40	0.29 ± 0.25	<0.01	1.00 ± 0.07	1.09 ± 0.48	1.00
miR-27b	1.00 ± 0.35	0.15 ± 0.06	<0.01	1.00 ± 0.36	0.75 ± 0.37	0.34
	**Liver**			**Lung**		
	**Control**	**KO**	***p* value**	**Control**	**KO**	***p* value**
miR-23a	1.00 ± 0.45	0.90 ± 0.29	0.89	1.00 ± 0.89	0.99 ± 0.80	0.69
miR-23b	1.00 ± 0.33	0.83 ± 0.25	0.34	1.00 ± 0.75	1.13 ± 0.83	0.89
miR-24	1.00 ± 0.47	0.94 ± 0.23	0.89	1.00 ± 0.85	1.07 ± 0.80	0.49
miR-27a	1.00 ± 0.53	0.61 ± 0.23	0.34	1.00 ± 0.97	0.98 ± 0.90	0.89
miR-27b	1.00 ± 0.51	0.65 ± 0.14	0.34	1.00 ± 1.11	0.95 ± 0.83	0.69
	**Muscle**					
	**Control**	**KO**	***p* value**			
miR-23a	1.00 ± 0.33	1.09 ± 0.70	0.69			
miR-23b	1.00 ± 0.29	1.02 ± 0.56	0.89			
miR-24	1.00 ± 0.29	1.01 ± 0.59	0.69			
miR-27a	1.00 ± 0.56	0.94 ± 0.68	1.00			
miR-27b	1.00 ± 0.41	1.00 ± 0.59	1.00			

**Table 2:** Expression of miR-23a/b clusters in 12-month-old mice. Expression of miR-23a/b cluster microRNAs in the corpus callosum, brain gray matter, white matter and gray matter of the spinal cord at 12 months of age. Mean ± standard deviation of expression relative to mean expression in control mice. Statistical differences between control mice and miR-23a/b cluster knockout (KO) mice were calculated.

	Brain Corpus Callosum			Brain Gray Matter		
	Control	KO	*p* Value	Control	KO	*p* Value
miR-23a	1.00 ± 0.68	0.12 ± 0.04	<0.01	1.00 ± 0.34	0.90 ± 0.31	0.69
miR-23b	1.00 ± 0.66	0.11 ± 0.03	<0.01	1.00 ± 0.14	0.91 ± 0.39	0.69
miR-24	1.00 ± 0.67	0.13 ± 0.06	<0.01	1.00 ± 0.09	1.10 ± 0.48	1.00
miR-27a	1.00 ± 0.83	0.07 ± 0.03	<0.01	1.00 ± 0.27	0.96 ± 0.35	0.89
miR-27b	1.00 ± 0.98	0.06 ± 0.02	<0.01	1.00 ± 0.23	1.01 ± 0.40	1.00
	**Spinal cord white matter**			**Spinal cord gray matter**		
	**Control**	**KO**	***p* value**	**Control**	**KO**	***p* value**
miR-23a	1.00 ± 0.49	0.32 ± 0.12	0.03	1.00 ± 0.34	0.95 ± 0.33	1.00
miR-23b	1.00 ± 0.35	0.23 ± 0.11	<0.01	1.00 ± 0.49	0.77 ± 0.43	0.89
miR-24	1.00 ± 0.40	0.39 ± 0.20	0.02	1.00 ± 0.39	1.11 ± 0.33	0.89
miR-27a	1.00 ± 0.39	0.38 ± 0.19	<0.01	1.00 ± 0.38	1.08 ± 0.19	0.89
miR-27b	1.00 ± 0.76	0.35 ± 0.32	0.03	1.00 ± 0.56	0.62 ± 0.42	0.49
	**Liver**			**Lung**		
	**Control**	**KO**	***p* value**	**Control**	**KO**	***p* value**
miR-23a	1.00 ± 0.13	1.06 ± 0.23	0.69	1.00 ± 0.17	1.03 ± 0.22	0.89
miR-23b	1.00 ± 0.41	0.93 ± 0.20	0.89	1.00 ± 0.61	1.64 ± 0.83	0.69
miR-24	1.00 ± 0.41	1.13 ± 0.36	0.34	1.00 ± 0.38	1.04 ± 0.29	0.89
miR-27a	1.00 ± 0.44	0.91 ± 0.30	1.00	1.00 ± 0.42	1.12 ± 0.49	0.89
miR-27b	1.00 ± 0.23	0.85 ± 0.22	0.69	1.00 ± 0.43	0.95 ± 0.24	1.00
	**Muscle**					
	**Control**	**KO**	***p* value**			
miR-23a	1.00 ± 0.32	1.27 ± 0.34	0.34			
miR-23b	1.00 ± 0.42	1.23 ± 0.30	0.49			
miR-24	1.00 ± 0.40	1.34 ± 0.27	0.34			
miR-27a	1.00 ± 0.41	1.04 ± 0.38	1.00			
miR-27b	1.00 ± 0.43	0.95 ± 0.69	0.69			

## Data Availability

The data that support the findings of this study are available from the corresponding author upon reasonable request.
